# Admission EEG findings in diverse paediatric cerebral malaria populations predict outcomes

**DOI:** 10.1186/s12936-018-2355-9

**Published:** 2018-05-22

**Authors:** Douglas G. Postels, Xiaoting Wu, Chenxi Li, Peter W. Kaplan, Karl B. Seydel, Terrie E. Taylor, Youssef A. Kousa, Richard Idro, Robert Opoka, Chandy C. John, Gretchen L. Birbeck

**Affiliations:** 1 International Neurologic and Psychiatric Epidemiology Program, Michigan State University, 909 Fee Road, 324 West Fee Hall, East Lansing, MI 48824 USA; 2 Department of Epidemiology and Biostatistics, Michigan State University, 909 Fee Road, Room B601, East Lansing, MI 48824 USA; 3 Department of Neurology, Johns Hopkins University, 4940 Eastern Avenue, Baltimore, MD 21224 USA; 4 Blantyre Malaria Project, University of Malawi College of Medicine, Blantyre, Malawi; 5 Department of Osteopathic Medical Specialties, College of Osteopathic Medicine, Michigan State University, East Lansing, MI 48824 USA; 6 Department of Neurology, Children’s National Health System, 111 Michigan Avenue NW, Washington, DC 20010 USA; 7 Department of Paediatrics and Child Health, Makerere University College of Health Sciences, Kampala, Uganda; 8 Indiana University School of Medicine, 1044 W. Walnut Street, Rm 402-D, Indianapolis, IN 46202 USA; 9 Epilepsy Division, Department of Neurology, University of Rochester, 265 Crittenden Blvd, CU 420694, Rochester, NY 14642 USA; 10 UTH Neurology Research Office, Nationalist Rd, PO Box UTH 11, Lusaka, Zambia

**Keywords:** Malaria, Paediatrics, Africa, Malawi, Uganda

## Abstract

**Background:**

Electroencephalography at hospital presentation may offer important insights regarding prognosis that can inform understanding of cerebral malaria (CM) pathophysiology and potentially guide patient selection and risk stratification for future clinical trials. Electroencephalogram (EEG) findings in children with CM in Uganda and Malawi were compared and associations between admission EEG findings and outcome across this diverse population were assessed. Demographic, clinical and admission EEG data from Ugandan and Malawian children admitted from 2009 to 2012 with CM were gathered, and survivors assessed for neurological abnormalities at discharge.

**Results:**

281 children were enrolled (Uganda n = 122, Malawi n = 159). The Malawian population was comprised only of retinopathy positive children (versus 72.5% retinopathy positive in Uganda) and were older (4.2 versus 3.7 years; p = 0.046), had a higher HIV prevalence (9.0 versus 2.8%; p = 0.042), and worse hyperlactataemia (7.4 versus 5.2 mmol/L; p < 0.001) on admission compared to the Ugandan children. EEG findings differed between the two groups in terms of average voltage and frequencies, reactivity, asymmetry, and the presence/absence of sleep architecture. In univariate analyses pooling EEG and outcomes data for both sites, higher average and maximum voltages, faster dominant frequencies, and retained reactivity were associated with survival (all p < 0.05). Focal slowing was associated with death (OR 2.93; 95% CI 1.77–7.30) and a lower average voltage was associated with neurological morbidity in survivors (p = 0.0032).

**Conclusions:**

Despite substantial demographic and clinical heterogeneity between subjects in Malawi and Uganda as well as different EEG readers at each site, EEG findings on admission predicted mortality and morbidity. For CM clinical trials aimed at decreasing mortality or morbidity, EEG may be valuable for risk stratification and/or subject selection.

## Background

Cerebral malaria (CM) is defined as an otherwise unexplained coma in a patient with malaria parasitaemia [1]. Despite improvements in anti-malarial treatment regimens, the mortality rate in tertiary care centres remains 15–25% and approximately one-third of survivors are left with neurological sequelae at hospital discharge including developmental or behavioural abnormalities, and epilepsy [2, 3]. Single-site studies have suggested that admission electroencephalogram (EEG) may offer important insights into both prognosis [4, 5] and the pathophysiology of CM [2]. Several phase II and III clinical trials aimed at improving survival and neurologic outcomes from CM are ongoing or have been recently completed [6–9]. Future phase III clinical trials could potentially benefit from validation of a non-invasive biomarker that allows identification of children at high risk of death. Such a marker might allow for subject enrichment during enrollment or stratification of collected data.

EEG is not routinely available across Africa. Previously published single-site case series have reported a number of abnormalities in the EEGs of children with CM including diffuse slowing, atypical sleep elements (spindles and vertex waves), and epileptiform activity [5, 10, 11]. In a case series of 65 Kenyan children with CM, a slower background frequency was significantly associated with an increased risk of death [5]. Burst-suppression was found in a higher proportion of children who died compared to those who lived, but the numbers of children with burst-suppression was small and the differences in proportions between outcome groups were not statistically significantly different. A report from Malawi identified a lack of variability and reactivity to painful stimulation as a risk factor for mortality [12]. A recently published study of acute magnetic resonance imaging and EEG findings in 168 Malawian children with CM determined that a slower background frequency was significantly associated with increased mortality risk [4]. No multi-site studies of EEG in CM have been published.

The epidemiology of CM is rapidly evolving due to climate change and successful control programs resulting in some regions with decreased disease burden, countered by other regions where incidence is constant or increasing [1]. Definitive clinical trials aimed at decreasing malaria morbidity and mortality will likely require multiple study sites both to assure access to adequate numbers of eligible children as well as improve the generalizability of results. To explore the potential value of EEG for subject selection or stratification in future clinical trials, admission EEG factors were compared between the two study sites, and the associations of these factors with death and neurodisability in Malawian and Ugandan children determined. This study’s goal was to identify EEG factors associated with death or neurological disability that could potentially inform future clinical trials. Identification of EEG changes associated with mortality and morbidity that might allow further insight into the underlying pathophysiology of death and disability in children with CM were assessed.

## Methods

Clinical and admission EEG data in consecutively admitted children with CM from Uganda and Malawi were gathered. Mulago Hospital is a large (1600 bed) Ugandan public tertiary care facility located in the country’s capital, Kampala. Queen Elizabeth Central Hospital (QECH) is the largest public health care facility in Malawi and located in the country’s commercial capital of Blantyre. Both hospitals are home to independent long-standing studies of severe malaria pathogenesis and natural history. Children 6 months to 15 years old, admitted to either Mulago or QECH with a clinical diagnosis of CM (Blantyre coma score ≤ 2, *Plasmodium falciparum* on peripheral blood smear, and no other known aetiology of coma) between December 2008 and January 2012 were eligible for inclusion. All children included in this sub-study were recruited for comprehensive projects studying CM pathogenesis and clinical epidemiology.

Nurse to patient ratios were high in both countries (one nurse to two patients or occasionally 1:1)—substantially higher than is the standard of routine care. In Malawi, children were hospitalized on a paediatric research ward dedicated to the study of children in coma, while in Uganda they were admitted to the high acuity ward (paediatric intensive care unit) with dedicated study nurses.

EEGs were recorded within 12 h of admission at both sites. All children were comatose (Blantyre Coma Score ≤ 2) at the time of recording. Malawi used a Ceegraph digital machine (BioLogic/Natus, Pleasanton, California) and Uganda an XLTEC 32 channel machine (Natus Medical Corporation, Pleasanton, California). EEG technicians used the international 10–20 system for electrode placement. Recording duration was 30 min or more, unless the patient became medically unstable or was otherwise unable to tolerate the procedure. EEG interpretations used for patient care were performed during hospitalization by study and non-study personnel to assess for non-convulsive status epilepticus or electrographic seizures. Research interpretations used for this study were performed after hospital discharge by a paediatric neurologist (DP, Uganda) or an epileptologist (GB and PK, Malawi) using Persyst Insight II (Persyst corporation, Prescott, Arizona). Interpreters were blinded to the child’s outcome. Interpretations were scored on a standardized form with predetermined definitions for each finding. These included: a determination of average background frequency; average and maximal voltages (excluding vertex sharp waves); the absence or presence of spontaneous EEG variability; the absence or presence of sleep spindles; the absence or presence of vertex waves; a quantitation of the proportion of epochs with sleep spindles (0, 1–25%, 26–50%, 51–75%, > 75%); identification and characterization of epileptiform activity; and determination of EEG reactivity. EEG technicians assessed reactivity by application of a painful stimulus (usually a sternal rub) to determine if changes in frequency or amplitude occurred. Previous experience with interpretation of EEGs in children with CM led us to characterize two types of reactivity, paradoxical or normal. EEG interpreters defined paradoxical reactivity as slowing in frequency or decrease in amplitude by more than 50% that occurred after application of a painful stimulus. Normal reactivity was defined as an increase in frequency or amplitude by more than 50% that occurred after stimulation. An electrographic seizure was defined as a rhythmic repetitive spike-wave discharge with an electrical field that evolved in frequency, morphology, or amplitude over at least 10 s. Although several children had multiple EEGs performed during their hospital stay, only the first EEG obtained was used in this analysis as this would be the one most relevant for informing future clinical trials.

During hospitalization, clinical seizures were treated first with intravenous diazepam or intramuscular paraldehyde. If ictal phenomena were recurrent or prolonged, phenobarbital was added. Breakthrough seizures on phenobarbital were treated by adding phenytoin. Clinical complications were treated as they developed. Continuous EEG monitoring was not performed in either centre.

In the children who survived, outcome was determined at hospital discharge by parent interview and a neurological exam; examinations were performed by physicians in Uganda and by a physician or clinical officer in Malawi. Clinical officers are paramedical professionals who undergo 3 years of technical training after secondary school. They are largely trained to use an algorithmic approach to patient care for conditions commonly seen in their region. They are not explicitly trained to conduct a detailed neurologic assessment.

Children whose parents stated they were not back to normal (new motor, cognitive, or behavioural abnormalities), or children found to have new neurological signs on examination were classified as ‘alive with sequelae’. Those with normal neurological exams and whose parents reported they were back to baseline were classified as ‘normal’. Across institutions, there were no further specific criteria to determine whether a child should be classified as normal versus alive with sequelae.

### Statistical analysis

Demographic factors, clinical characteristics and discharge outcomes of the study population were described using means (and standard deviations) or proportions. To evaluate differences between study site populations, comparisons were made in continuous and categorical EEG variables in Malawi and Uganda. Wilcoxon rank sum tests were used for comparisons of continuous data. Pearson’s Chi square tests were used for categorical data where all cell counts were ≥ 5; Fisher’s exact tests were used to analyse categorical data with cell counts less than 5. Pooling data from Uganda and Malawi, univariate analyses of continuous EEG variables (e.g. frequency, amplitude) in children of differing outcomes were compared using Wilcoxon rank sum tests. Categorical EEG variables (e.g. asymmetry, reactivity, variability, presence or absence of electrographic seizures) in children of differing outcomes were compared using Pearson’s Chi square or Fisher’s exact test, as appropriate. A two-sided p < 0.05 was considered a statistically significant difference between groups.

## Results

During the study period, 281 EEGs were performed and analysed, 122 from Uganda and 159 from Malawi. Tables 1 and 2 compare demographic, clinical, EEG and outcomes data between the two sites. The Malawian population was comprised only of retinopathy positive children (versus 72.5% in Uganda) and were older (4.2 versus 3.7 years; p = 0.046), had a higher HIV prevalence (9.0 versus 2.8%; p = 0.042), and worse hyperlactataemia (7.4 versus 5.2 mmol/L; p < 0.001) on admission than Ugandan children (Tables 1 and 2). In Uganda, 21 children (17.2%) died and 44 of the 100 survivors (44.0%) had neurological sequelae at hospital discharge. (In one Ugandan survivor, the presence or absence of neurological sequelae was unknown.) In Malawi, outcomes were better: 22 children (13.8%) died, and 11.3% (16 of the 137 survivors) had neurological sequelae. There was no statistically significant difference in the mortality rates between Malawi and Uganda (p = 0.408). Rates of morbidity in survivors were significantly higher in Uganda than Malawi (p = 0.002).

**Table 1:** Comparison of clinical and EEG characteristics (continuous variables) between Malawian and Ugandan children with cerebral malaria (value ± SD) (denominators vary due to missing data)

Variable	Uganda (N = 122)		Malawi (N = 159)		p value for difference
	N	Mean ± SD	N	Mean ± SD	
Age (years)	122	3.75 ± 1.95	159	4.23 ± 2.27	0.0463
Glucose (mg/dL)	119	6.6 ± 3.4	159	7.1 ± 3.7	0.8200
Parasitemia (parasites/µL) (geometric mean)	116	33,288.5	148	33,239.2	0.9961
Lactate (mmol/L)	117	5.2 (4.0)	159	7.4 (4.1)	< 0.0001
Blantyre Coma Score (n/N, %)					0.91
0		9 (7.4)		12 (7.6)	
1		57 (46.7)		78 (49.1)	
2		56 (45.9)		69 (43.4)	
Average voltage (µV)	117	65.77 ± 31.84	151	109.1 ± 55.17	< 0.0001
Maximum voltage (µV)	117	198.0 ± 73.16	152	209.9 ± 88.62	0.3866
Dominant frequency (Hz)	115	1.93 ± 0.73	148	2.34 ± 1.09	0.0132

**Table 2:** Comparison of clinical and EEG characteristics (categorical variables) between Malawian and Ugandan children with cerebral malaria (denominators vary due to missing data)

Variable	Uganda (n/N, %)	Malawi (n/N, %)	p value for difference
Retinopathy positive	87/120 (72.5)	159/159 (100)	–
HIV reactivity	3/109 (2.8)	13/144 (9.0)	0.0423
Variability (% with)	73/119 (61.3)	78/159 (49.1)	0.0418
Asymmetry (% with)	15/122 (12.3)	49/159 (30.8)	0.0002
Electrographic seizures (% with)	9/122 (7.4)	22/159 (13.8)	0.0867
Spindles (% with)	71/116 (61.2)	65/159 (40.9)	0.0009
Vertex sharp waves (% with)	56/118 (47.5)	39/159 (25.0)	< 0.0001
Slowing location (% focal)	5/117 (4.2)	19/159 (11.9)	0.0240
Normal reactivity (% with increase in voltage and/or frequency with stimulus)	4/113 (3.5)	41/153 (26.8)	< 0.0001
Paradoxical reactivity (% with decrease in voltage and/or frequency with stimulus)	40/113 (35.4)	27/153 (17.6)	

There were multiple statistically significant differences in admission EEGs when comparing Malawian and Ugandan children (Tables 1 and 2). Average voltage was lower and frequencies were slower in EEGs from Uganda compared to those from Malawi. EEG variability, sleep spindles, and vertex waves were significantly more common in Ugandan children, while EEGs from Malawian children were more likely to show asymmetry. There was no statistically significant difference in the proportion of children with electrographic seizures in the two locations.

Tables 3 and 4 delineate the univariate analyses of pooled EEG findings associated with mortality. Death was associated with a lower average voltage (93.8 µV in survivors compared to 69.9 µV in those who died, p = 0.0032), lower maximum voltage (210.6 µV in survivors compared to 171.1 µV in those who died, p = 0.0089), and slower dominant frequency (2.2 Hz in survivors compared to 1.8 Hz in those who died, p = 0.0030). Focal slowing was associated with death (OR 2.93; 95% CI 1.77–7.30) and reactivity with survival (OR for death 0.37; 95% CI 0.18–0.79).

**Table 3:** Univariate analysis: Comparison of continuous clinical and EEG variables by outcome (mortality) (mean ± SD)

Country	Clinical or EEG variable	Alive (N = 101)	Died (N = 21)	p value
Uganda	Age (years)	3.8 ± 1.9	3.6 ± 2.1	0.77
	Glucose (mg/dL)	6.8 ± 3.3	5.6 ± 3.6	0.2622
	Parasitemia (parasites/µL) [geometric mean (coefficient of variation)]	30,315.4 (21.9)	53,669.7 (6.5)	0.3463
	Lactate (mmol/L)	4.9 ± 3.9	6.7 ± 4.1	0.0467
	Average voltage (µV)	68.9 ± 31.9	50.5 ± 27.4	0.018
	Maximum voltage (µV)	206.3 ± 71.0	158.0 ± 71.9	0.007
	Dominant frequency (Hz)	2.0 ± 0.7	1.6 ± 0.9	0.02

Country	Clinical or EEG variable	Alive (N = 137)	Died (N = 22)	p value
Malawi	Age (years)	4.3 ± 2.3	4.1 ± 2.2	0.70
	Glucose (mg/dL)	7.1 ± 3.6	7.4 ± 4.1	0.8675
	Parasitemia (parasites/µL) [geometric mean (coefficient of variation)]	31,053.5 (25.4)	50,153.1 (13.6)	0.4189
	Lactate (mmol/L)	7.2 ± 4.1	8.6 ± 3.8	0.0827
	Average voltage (µV)	112.2 ± 54.3	89.3 ± 58.2	0.08
	Maximum voltage (µV)	213.8 ± 88.4	184.3 ± 88.2	0.17
	Dominant frequency (Hz)	2.4 ± 1.1	1.9 ± 0.9	0.05

Country	Clinical or EEG variable	Alive (N = 238)	Died (N = 43)	p value
Uganda + Malawi	Age (years)	4.0 ± 2.1	3.8 ± 2.1	0.3459
	Glucose (mg/dL)	7.0 ± 3.5	6.5 ± 4.0	0.5049
	Parasitemia (parasites/µL) [geometric mean (coefficient of variation)]	30,731.7 (23.5)	51,793.8 (9.0)	0.2168
	Lactate (mmol/L)	6.2 ± 4.2	7.7 ± 4.0	0.0170
	Average voltage (µV)	93.8 ± 50.8	69.9 ± 49.0	0.0032
	Maximum voltage (µV)	210.6 ± 81.4	171.1 ± 80.6	0.0089
	Dominant frequency (Hz)	2.2 ± 1.0	1.8 ± 0.9	0.0030

**Table 4:** Comparison of categorical demographic and EEG variables by outcome (mortality) (OR, 95% CI)

Country	Clinical or EEG variable	Children with variable who were alive (N, %)	Children with variable who died (N, %)	Odds ratio^a^	95% confidence interval	p value
Uganda	Total N (122)	101	21			
	Retinopathy positive	71 (70.3)	16 (73.2)	1.63	0.5, 5.3	0.41
	HIV reactivity	3 (3.0)	0 (0.0)			
	Variability	62 (61.4)	11 (52.4)	0.64	0.25, 1.65	0.35
	Asymmetry	13 (12.9)	2 (9.5)	0.71	0.15, 3.42	0.67
	Electrographic seizures (present)	8 (7.9)	1 (4.8)	0.58	0.07, 4.9	0.62
	Spindles (present)	60 (59.4)	11 (52.4)	0.64	0.25, 1.66	0.36
	Vertex sharp waves	47 (46.5)	9 (42.9)	0.79	0.31, 2.07	0.64
	Focal slowing	3 (2.9)	3 (14.3)	5.27	0.98, 28.3	0.052
	Reactivity (normal + paradoxical)	40 (39.6)	4 (19.1)	0.306	0.09, 0.98	0.046
	Normal reactivity (increase in voltage and/or frequency with stimulus)	37 (36.6)	3 (14.3)	1.02	0.09, 10.46	0.55
	Paradoxical reactivity (decrease in voltage and/or frequency with stimulus)	3 (2.97)	1 (4.76)	0.25	0.07, 0.91	0.09
Malawi	Total N (159)	137	22			
	Retinopathy positive	137 (100)	22 (100)			
	HIV reactivity	12 (9.7)	1 (5.0)	0.49	0.06, 4.0	0.5065
	Variability	71 (51.8)	7 (31.8)	0.43	0.17, 1.13	0.087
	Asymmetry	40 (29.2)	9 (40.9)	1.68	0.66, 4.23	0.27
	Electrographic seizures present	16 (11.7)	6 (27.3)	2.84	0.97, 8.3	0.06
	Spindles present	58 (42.3)	7 (31.8)	0.64	0.24, 1.66	0.35
	Vertex sharp waves	37 (27.0)	2 (9.1)	0.27	0.06, 1.21	0.09
	Focal slowing (focal versus gen)	14 (10.2)	5 (22.7)	2.58	0.83, 8.08	0.10
	Reactivity (normal + paradoxical)	62 (45.3)	6 (27.3)	0.45	0.17, 1.2	0.12
	Normal reactivity (increase in voltage and/or frequency with stimulus)	37 (27.0)	4 (18.2)	0.51	0.16, 1.63	0.77
	Paradoxical reactivity (decrease in voltage and/or frequency with stimulus)	25 (18.2)	2 (9.1)	0.37	0.08, 1.75	0.42
Uganda + Malawi	Total N (281)	238	43			
	Retinopathy positive	208 (87.4)	38 (88.4)	1.32	0.44,3.98	0.62
	HIV reactivity	15 (6.7)	1 (3.5)	0.49	0.06, 3.91	0.5072
	Variability	133 (55.9)	18 (41.9)	0.552	0.29,1.07	0.077
	Asymmetry	53 (22.3)	11 (25.6)	1.20	0.57,2.54	0.634
	Electrographic seizures present	24 (10.1)	7 (16.3)	1.73	0.69,4.32	0.24
	Spindles present	118 (49.6)	18 (41.9)	0.696	0.36,1.34	0.28
	Vertex sharp waves	84 (35.3)	11 (25.6)	0.614	0.29,1.28	0.19
	Focal slowing	17 (7.1)	8 (18.6)	2.93	1.76,7.3	0.02
	Reactivity (normal + paradoxical)	102 (42.9)	10 (23.3)	0.374	0.18,0.79	0.010
	Reactivity (increase in voltage and/or frequency with stimulus)	40 (16.8)	5 (11.6)	0.477	0.17,1.31	0.79
	Paradoxical reactivity (decrease in voltage and/or frequency with stimulus)	62 (26.1)	5 (11.6)	0.308	0.11,0.83	0.13

^a^Odds of death of children having the EEG feature divided by the odds of death of children without the EEG feature

A univariate analysis of pooled EEG findings associated with neurological morbidity in survivors revealed that a higher average voltage was associated with a normal neurological outcome in survivors (101.9 µV in those with a normal outcome versus 70.1 µV in those with neurological sequelae, p < 0.0001) (Tables 5 and 6).

**Table 5:** Univariate analysis: comparison of continuous clinical and EEG variables by outcome (neurological morbidity in survivors) (mean ± SD)

Country	Clinical or EEG variable	Alive and normal (N = 56)	Alive with sequelae (N = 44)	p value
Uganda	Age (years)	3.8 ± 1.7	3.8 ± 2.2	0.5312
	Glucose (mg/dL)	7.2 ± 3.3	6.4 ± 3.4	0.1376
	Parasitemia (parasites/µL) [geometric mean (coefficient of variation)]	31,931.6 (23.3)	31,434.9 (18.7)	0.9755
	Lactate (mmol/L)	4.6 ± 3.7	5.3 ± 4.2	0.4449
	Average voltage (µV)	74.0 ± 32.1	63.6 ± 30.8	0.0723
	Maximum voltage (µV)	213.8 ± 71.2	199.1 ± 70.3	0.3809
	Dominant frequency (Hz)	2.0 ± 0.7	2.0 ± 0.6	0.9210

Country	Clinical or EEG variable	Alive and normal (N = 121)	Alive with sequelae (N = 16)	p value
Malawi	Age (years)	4.3 ± 2.3	3.5 ± 1.9	
	Glucose (mg/dL)	6.9 ± 2.9	8.3 ± 7.0	0.6468
	Parasitemia (parasites/µL) [geometric mean (coefficient of variation)]	28,148.2 (27.4)	73,486.0 (10.4)	0.1988
	Lactate (mmol/L)	7.0 ± 4.0	8.4 ± 4.8	0.3023
	Average voltage (µV)	114.5 ± 54.2	91.5 ± 52.6	0.2178
	Maximum voltage (µV)	217.4 ± 86.3	180.0 ± 103.1	0.1185
	Dominant frequency (Hz)	2.4 ± 1.1	2.1 ± 1.3	0.1550

Country	Clinical or EEG variable	Alive and normal (N = 177)	Alive with sequelae (N = 60)	p value
Uganda + Malawi	Age (years)	4.2 ± 2.2	3.7 ± 2.1	0.0723
	Glucose (mg/dL)	7.0 ± 3.0	6.9 ± 4.7	0.1999
	Parasitemia (parasites/µL) [geometric mean (coefficient of variation)]	29,312.6 (25.57)	38,422.1 (16.66)	0.4876
	Lactate (mmol/L)	6.3 ± 4.0	6.1 ± 4.5	0.5321
	Average voltage (µV)	101.9 ± 51.9	70.1 ± 38.4	< 0.0001
	Maximum voltage (µV)	216.3 ± 81.8	194.6 ± 78.5	0.0943
	Dominant frequency (Hz)	2.3 ± 1.0	2.0 ± 0.8	0.0524

**Table 6:** Comparison of categorical demographic and EEG variables by outcome (neurological morbidity in survivors) (OR, 95% CI)

Country	Clinical or EEG variable	Children with variable who were alive with sequelae (N, %)	Children with variable who were alive without sequelae (N, %)	Odds ratio^a^	95% confidence interval	p value
Uganda	Total N (101)					
	Retinopathy positive	32 (72.7)	38 (69.1)	1.19	0.49, 2.86	0.6930
	HIV reactivity	3 (6.9)	0			
	Variability	28 (63.6)	34 (64.2)	0.98	0.43, 2.25	0.9581
	Asymmetry	4 (9.1)	9 (16.1)	0.52	0.15, 1.83	0.3089
	Electrographic seizures (present)	3 (6.8)	5 (8.9)	0.75	0.17, 3.31	0.7002
	Spindles (present)	26 (61.9)	34 (64.2)	0.91	0.39, 2.10	
	Vertex sharp waves	22 (51.2)	25 (47.2)	1.17	0.52, 2.63	0.6972
	Focal slowing	0	2 (3.8)			
	Reactivity (normal + paradoxical)	15 (37.5)	25 (49.0)	0.62	0.27, 1.45	0.2730
	Normal reactivity (increase in voltage and/or frequency with stimulus)	2 (5.0)	1 (1.9)	2.08	0.18, 24.40	0.4128
	Paradoxical reactivity (decrease in voltage and/or frequency with stimulus)	13 (32.5)	24 (47.1)	0.56	0.24, 1.35	0.1895
Malawi	Total N (137)					
	HIV reactivity	1 (7.1)	11 (10.0)	0.69	0.08, 5.81	0.7347
	Variability	7 (43.8)	64 (52.9)	0.69	0.24, 1.98	0.4932
	Asymmetry	7 (43.8)	33 (27.3)	2.07	0.72, 6.02	0.1797
	Electrographic seizures present	6 (37.5)	10 (8.3)	6.7	2.0, 22.1	0.002
	Spindles present	5 (31.3)	53 (43.8)	0.58	0.19, 1.78	0.3438
	Vertex sharp waves	1 (6.3)	36 (29.8)	0.16	0.02, 1.24	0.0788
	Focal slowing (focal versus gen)	4 (25.0)	10 (8.3)	3.7	1.01, 13.6	0.0491
	Reactivity (normal + paradoxical)	6 (37.5)	56 (48.3)	0.64	0.22, 1.89	0.4209
	Normal Reactivity (increase in voltage and/or frequency with stimulus)	5 (31.3)	32 (27.6)	0.94	0.29, 2.98	0.3837
	Paradoxical reactivity (decrease in voltage and/or frequency with stimulus)	1 (6.3)	24 (20.7)	0.25	0.03, 2.06	0.2025
Uganda + Malawi	Total N (281)					
	Retinopathy positive	48 (80.0)	159 (90.3)	0.43	0.19, 0.96	0.0390
	HIV reactivity	4 (7.0)	11 (6.6)	1.06	0.33, 3.48	0.9190
	Variability	35 (58.3)	98 (56.3)	1.09	0.59, 1.97	0.7862
	Asymmetry	11 (18.3)	42 (23.7)	0.72	0.34, 1.51	0.3875
	Electrographic seizures present	9 (15.0)	15 (8.5)	1.91	0.79, 4.62	0.1529
	Spindles present	31 (53.5)	87 (50.0)	1.15	0.63, 2.08	0.6493
	Vertex sharp waves	23 (38.9)	61 (35.1)	1.18	0.64, 2.18	0.5875
	Focal slowing	4 (6.8)	12 (6.9)	0.98	0.30, 3.17	0.9756
	Reactivity (normal + paradoxical)	21 (37.5)	81 (48.5)	0.64	0.34, 1.19	0.1543
	Reactivity (increase in voltage and/or frequency with stimulus)	7 (12.5)	33 (19.8)	0.52	0.21, 1.29	0.2856
	Paradoxical reactivity (decrease in voltage and/or frequency with stimulus)	14 (25.0)	48 (28.7)	0.72	0.35, 1.46	0.9846

^a^Odds of disability of children having the EEG feature divided by the odds of disability of children without the EEG feature

## Discussion

Despite the substantial noise of demographic and clinical heterogeneity between subjects in Malawi and Uganda as well as different EEG readers at each site, there is a clear signal that EEG findings on admission predict mortality and morbidity.

Admission EEG findings in children with CM from Uganda differ from those recorded in Malawi. Possible explanations for these differences may be specific to EEG (study technique, interpreter effect), intrinsic to the nature of any multi-centre study (studies obtained at different time points, different inclusion criteria) or could represent true differences between the Malawian and Ugandan paediatric CM populations.

EEG interpretation is a subjective process with no currently available tools for objective, reliable quantification of frequency, voltage, or any of the variables captured in this study. Although automated tools are available to aid interpretation, these are sensitive to artifact, creating both false positive and negative findings, when compared to human interpretation as the gold standard. Three interpreters reviewed the EEGs included here, which likely introduced variability into interpretations. Inter-rater correlation between EEG variables in often modest [13], but relying on a single reader limits external generalizability [14]. The imperative to improve EEG inter-rater reliability in the identification of triphasic waves, hypsarrhythmia and sleep architecture has recently resulted in efforts aimed at developing more standardized terminology and/or additional reader training for the identification of specific findings [15–18]. If EEG is to be used for stratification and/or risk adjustment in multi-site clinical trials of CM, improved standardization and/or development of validated sensitive and specific EEG interpretation software will be needed.

Despite these challenges, it is possible that the differences seen in admission EEGs between Malawian and Ugandan children represent intrinsic electrophysiological differences between populations. In terms of pathophysiological insights, many of the EEG findings described in this study are seen in other aetiologies of paediatric coma. EEG findings in children with non-malarial central nervous system infections are considered non-specific as to aetiology (e.g. viral versus bacterial) and consist of generalized delta waves [19], as was seen in these study subjects. This pattern implies that large areas of subcortical white matter are functionally abnormal [20]. Generalized slowing is seen more commonly in disorders primarily affecting white matter [21].

In a previous study of 201 children in coma in Italy, 26 of 77 children with central nervous system bacterial or viral infections had convulsions, though the authors did not clarify whether seizure activity was clinical, electrographic, or both [19]. Clinical seizures are very common in CM. In this study, non-continuous electrographic seizures captured on a 30 min EEG were documented in only 11.0% of subjects and were not associated with mortality or morbidity on pooled univariate analysis.

This study has several limitations. Continuous EEG monitoring was not performed, which likely led to a lack of sensitivity for ascertaining transient events. In paediatric traumatic brain injury, subclinical electrographic seizures are common, and the combination of clinical and electrographic seizures may occur in up to 77.3% of patients [14]. Studies of comatose children in paediatric intensive care units without restriction by coma aetiology reveal a 7% risk of subclinical electrographic seizures [22], a proportion similar to that seen in this study’s subjects. It is not possible to know if continuous EEG sampling would have modified the associations found here.

The analysis of EEG factors associated with neurological morbidity was complicated by a non-standard definition of this outcome across sites. For example, children with mild ataxia that was resolving were coded as “normal” in Malawi, but “abnormal” in Uganda. Future studies comparing EEG factors associated with neurological morbidity should standardize criteria to define neurosequelae. Children were classified as having neurological morbidity at discharge. Since neurological sequelae at discharge may clear over time, or epilepsy may appear after a delay of months, misclassification may have occurred. Since post-CM epilepsy can develop months after hospitalization, this study could not identify acute EEG characteristics that are predictors of later developing epilepsy. Future studies should consider ascertainment of admission EEG factors associated with long-term neurological disability. In younger patients, post-CM developmental disabilities may be difficult to detect and only become clear as the patient ages, when the survivor’s developmental milestones diverge from those of their unaffected peer group. Identification of EEG changes in children at higher risk of a long-term adverse outcome may allow focus of limited resources (physiotherapy, palliative care) to those at highest risk.

Retinopathy negative children were not represented in Malawi, as children from this site were enrolled in a parent study whose primary interest was retinopathy positive CM. Since one-third of children with clinical CM are retinopathy negative, the population from Malawi may not be representative of the clinical spectrum of children with this illness.

Identification of EEG factors associated with death and long-term adverse neurological outcome may allow insights into whether these outcomes share a common pathophysiology. Since seizures are considered primarily a gray matter-based abnormality, while slowing and decreased amplitudes are primary due abnormalities in the thalamo-cortical circuits (white matter based), it is possible that interventions successful in reducing rates of one outcome (mortality) may not lead to a change in the proportion of children with the other endpoint (e.g. an increase in the proportion of survivors with neurological disabilities).

## Conclusions

In children with CM, death is associated with patterns on admission EEG. Findings increasing the risk of death include a lower average voltage, lower maximum voltage, slower dominant frequencies, focal slowing, and a lack of reactivity. For CM clinical trials aimed at decreasing mortality, EEG may be valuable for risk stratification and/or subject selection.
